# Diabetic chorea as a neurological complication

**DOI:** 10.1002/ccr3.1579

**Published:** 2018-05-15

**Authors:** Tsuneaki Kenzaka, Moemi Fujikawa, Masanori Matsumoto

**Affiliations:** ^1^ Division of Community Medicine and Career Development Kobe University Graduate School of Medicine Kobe Japan; ^2^ Department of Internal Medicine Hyogo Prefectural Kaibara Hospital Tanba Japan

**Keywords:** diabetes mellitus, diabetic chorea, neurological complication

## Abstract

Diabetic chorea accompanies hyperglycemic states or drastic changes in blood glucose levels and involves the acute onset of unilateral or bilateral choreatic movements. Diabetes is sometimes discovered due to diabetic chorea, and thus, diabetic chorea is considered an important neurological complication.

The patient was a 57‐year‐old man being treated for bipolar disorder. He had been diagnosed with type 2 diabetes, but was not receiving drug therapy. A month before the examination, his depressive symptoms worsened because of stress at work. He was spending more time at home, mainly consuming soft drinks, sweet breads, and other foods that were high in sugar. One week before the admission to our hospital, his right arm began moving involuntarily. The symptoms worsened, so he came for outpatient examination. Blood test results were as follows: blood glucose, 573 mg/dL; HbA1c, 16.0%; calculated serum osmolality, 315 mOsm/kg; pH, 7.359; pCO_2_, 41.9 mm Hg; and bicarbonate ions, 23.1 mEq/L. He did not present with liver dysfunction or electrolyte imbalance or thyroid dysfunction, and was negative for urinary ketones. Head CT showed isodense regions in the left putamen (Figure 1). Video S1 shows the choreatic movements observed during inpatient observation. Glucose control improved with insulin therapy, and after about 1 week, the choreatic movements also improved. Based on the acute onset of unilateral choreatic movements that improved along with glucose control, the patient was diagnosed with diabetic chorea, which is also recognized as chorea, hyperglycemia, basal ganglia syndrome (C‐H‐BG).

**Figure 1:**
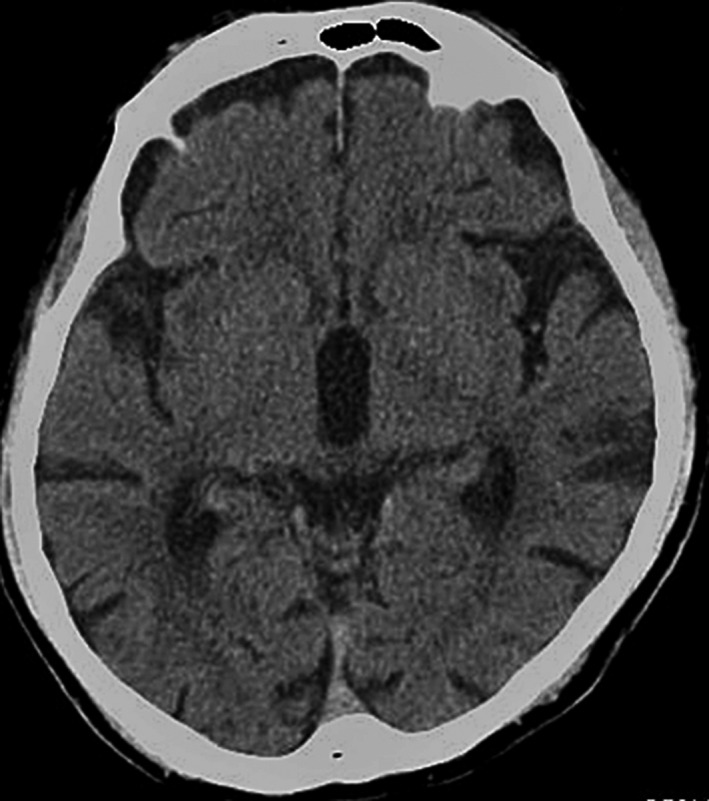
Isodensity to mild hyperdensity in the left putamen in head CT scan

## CONFLICT OF INTEREST

None of the authors have any financial interests to disclose, nor do they have any conflict of interests to declare.

## AUTHORS’ CONTRIBUTIONS

TK: managed redaction, correction, and reconstruction of the manuscript. MF: managed the case and redaction and correction of the manuscript. MK: assisted with clinical management of the case and correction of the manuscript. All authors read and approved the final manuscript.

## Supporting information


** **

